# Design of a High-Efficiency Multilayer Dielectric Diffraction Grating with Enhanced Laser Damage Threshold

**DOI:** 10.3390/nano12121952

**Published:** 2022-06-07

**Authors:** Duy Thanh Cu, Tien Dat Pham, Vu Tuan Hung Le, Meng Chi Li, Hung Pin Chen, Chien Cheng Kuo

**Affiliations:** 1 Department of Optics and Photonics/Thin Film Technology Center, National Central University, 300, Chung Da Rd., Chung Li, Taoyuan 32001, Taiwan; duythanh@g.ncu.edu.tw (D.T.C.); tiendat@g.ncu.edu.tw (T.D.P.); 2 Department of Applied Physics, Faculty of Physics and Engineering Physics, Ho Chi Minh City University of Science, 227 Nguyen Van Cu Street, Award 4, District 5, Ho Chi Minh City 748000, Vietnam; lvthung@hcmus.edu.vn; 3 Optical Sciences Center/Thin Film Technology Center, National Central University, 300, Chung Da Rd., Chung Li, Taoyuan 32001, Taiwan; mcli@dop.ncu.edu.tw; 4 National Applied Research Laboratories, Taiwan Instrument Research Institute, No. 20. R&D Rd. VI, Hsinchu Science Park, Hsinchu 30076, Taiwan; chbin@itrc.narl.org.tw

**Keywords:** multilayer dielectric grating, diffraction efficiency, high laser damage threshold

## Abstract

Diffraction gratings are becoming increasingly widespread in optical applications, notably in lasers. This study presents the work on the characterization and evaluation of Multilayer Dielectric Diffraction Gratings (MDG) based on the finite element method using Comsol MultiPhysics software. The optimal multilayer dielectric diffraction grating structure using a rectangular three-layer structure consisting of an aluminum oxide Al_2_O_3_ layer sandwiched between two silicon dioxide SiO_2_ layers on a multilayer dielectric mirror is simulated. Results show that this MDG for non-polarized lasers at 1064 nm with a significantly enhanced −1st diffraction efficiency of 97.4%, reaching 98.3% for transverse-electric (TE) polarization and 96.3% for transverse-magnetic (TM) polarization. This design is also preferable in terms of the laser damage threshold (LDT) because most of the maximum electric field is spread across the high LDT material SiO_2_ for TE polarization and scattered outside the grating for TM polarization. This function allows the system to perform better and be more stable than normal diffraction grating under a high-intensity laser.

## 1. Introduction

Laser technology is advancing at a breakneck pace, with extensive use in applications [1] such as material processing, scientific research, laser weaponry, and three-dimensional printing. The attempt to power-scale laser systems has centered on two different techniques for years [2]. One of the techniques is the improvement of each laser system component, such as the lasing mode [3], the pumping technology, and the development of near-diffraction suppression systems [4,5,6,7,8]. Nevertheless, this approach has significant drawbacks due to the nonlinearity effect [9], and the broadening of the spectrum for high-power lasers is unavoidable, severely limiting the output power of the laser channel [10,11].

Another technique for overcoming these drawbacks is to employ a multi-aperture beam combining method to produce high-quality composite beams. Coherent beam combining (CBC) and spectral beam combining (SBC) methods have been used to considerably increase the output of laser power [12]. A CBC system involves vectorially summing the outputs of multiple lasers by loading them into a spatial array and then phase-locking individual emitters to each other or to a common master oscillator. However, combining many lasers into a single coherent beam is challenging with only limited success [13]. Meanwhile, the SBC method combines many wavelength sources into a single output beam. In the SBC system, each source operates at a different wavelength. Therefore, the combined beam overlaps in the near and far fields without spatial interference.

Many research works have been conducted to discover the appropriate structure for the SBC method recently. Cho et al. [14] reported basic design methods and modal analysis for a near-Littrow grating with a high diffraction efficiency for both polarization forms in a given range of wavelengths. However, further enhancement in the diffraction efficiency required some modifications to the grating structure. As increasingly complicated and efficient diffractive structures were needed, the complexity of the grating structure quickly increased [15]. Depending on the desired optical functionality, the required grating surface patterns might have two, three, or even more layers. Li and Wang [16] employed a modal technique to build and evaluate a three-layer all-dielectric rectangular-groove transmission grating. The design exhibited the −first diffraction efficiency of more than 95% in the wavelength bandwidth range of 766–833 nm. This efficiency was significantly enhanced compared to those of single-layer and two-layer gratings.

Many researchers have devised different formalisms that can yield reliable computations of dielectric gratings. Compared with other methods, the finite element method (FEM) is a simpler and more efficient analytical tool for grating diffraction issues [17,18].

Alessi et al. reported a simulation using FEM to investigate the thermal-mechanical evolution of a single diffraction grating of a compressor [19]. Huang et al. [20] developed multilayer trapezoidal gratings, which comprise a layer of Hafnium dioxide HfO_2_ sandwiched between two SiO_2_ layers on a metal layer. This structure had a diffraction efficiency of 95.62% for TE polarization at an incidence angle of 53°. They employed the FEM to illustrate the normalized electric field intensity of the metal multilayer dielectric gratings (MDG) and discovered that the electric field focused on the interlayer HfO_2_ of the grating region. Nevertheless, their research had two limitations. Firstly, the metal used for this grating would exhibit losses of at least several percent due to absorption, particularly in power scaling, where such losses are completely undesirable [21]. Secondly, although the laser damage threshold (LDT) was improved, the increment still did not reach the optimal level because the electric field was still primarily focused on the HfO_2_ layer, which has an average LDT of 1.97 J/cm^2^ [22].

Therefore, multilayer dielectric mirrors are extensively utilized instead of metallic layers owing to their absorption-free properties [14,19,20,21]. As a result of replacing the metal with a dielectric multilayer dielectric mirror in our investigation, the diffraction efficiency is greatly boosted. The HfO_2_ material is substituted with a high LDT Al_2_O_3_ of 2.52 J/cm^2^ [22] in our study. Al_2_O_3_ is a material with a high refractive index and high LDT paired with a low refractive index SiO_2_ to provide a high diffraction efficiency and stability of MDG in high laser intensity environments. In addition, many rectangular gratings have been extensively studied using the photoresist mask method and ion beam etching method because of their ease of fabrication and high diffraction efficiency stability [23,24,25]. Consequently, utilizing gratings with a rectangular structure will result in great productivity.

The aim of the study is to use FEM to develop the structure of the grating in order to meet the criteria of high diffraction efficiency and LDT. Grating’s parameter computations are used to evaluate the diffraction efficiency and electric field distribution. Our study on the rectangular-groove three-layer grating on a multilayer dielectric mirror obtained after scanning data using the FEM demonstrates a considerable increase in the diffraction efficiency of the grating for TE and TM polarization. Simultaneously, most of the strongest electric fields for TE polarization are spread on the high LDT SiO_2_ layers and scattered outside the grating for TM polarization in the given grating model. This finding indicates that this design provides a significant improvement in LDT.

## 2. Materials and Methods

The diffraction equation in reflection is as follows [26]:

(1) $$n_{i}sin\theta_{m}=n_{i}sin\theta_{i}+m\frac{\lambda }{p}$$

where: *p* is the grating period, *n_i_* is the refractive index of the incident media, $\theta_{m}$ is the diffraction angle, $\theta_{i}\;$is the incident angle, m is the diffraction order, and λ is the wavelength of incident light.

A particular grating in which light is diffracted back toward the direction (i.e., $\theta_{m}$= $\theta_{i}$) is termed the Littrow mounting (or Littrow configuration). The grating equation is stated as follows [14]:

(2) $$-2n_{i}sin\theta_{m}=m\frac{\lambda }{p}$$

**Figure 1:**
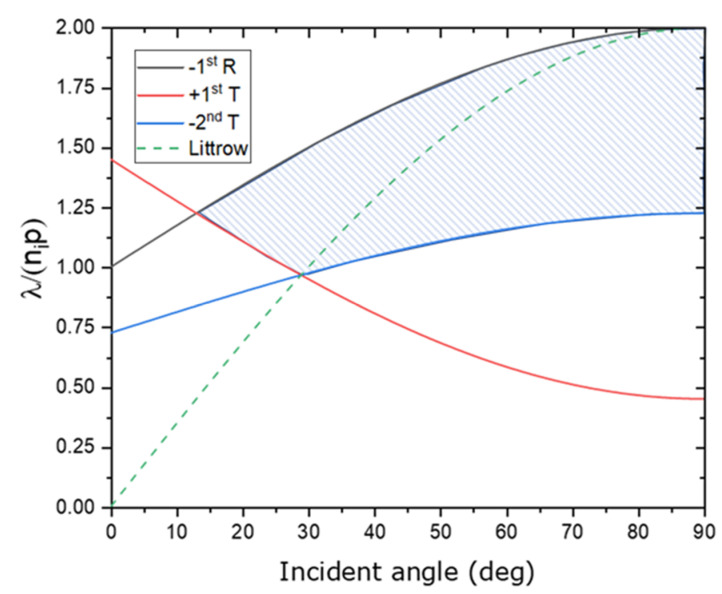
Dispersion of diffraction orders in reflection and transmission.

Figure 1 shows the dispersion of diffraction orders in reflection and transmission as a function of incident angle and (wavelength/period), $n_{i}$ is the refractive index of air (=1). Only the 0th and −1st orders in R and T exist in a dashed area, delimited by −1st R, +1st T, and −2nd T lines. The Littrow condition showed as a long-dashed line in which the −1st order diffracted back in the incident direction $\frac{\lambda }{n_{i}p}=2sin\theta_{i}$.

**Figure 2:**
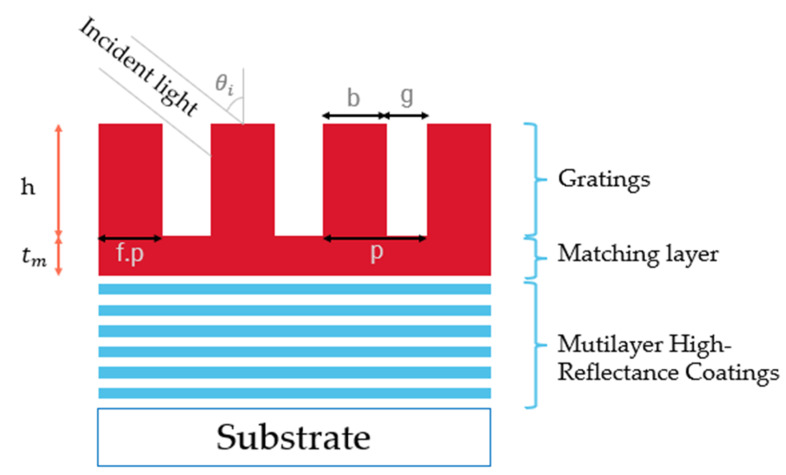
Schematic of MDG.

Hehl et al. [27] used the effective index of a diffraction grating to set the range of the grating period and incident angle. Diffraction orders may be used to represent the reflected and transmitted fields as a sum of plane waves. Based on their method, Figure 2 shows the range of diffraction orders as a function of the incident angle and grating period at a wavelength of 1064 nm. Gratings suitable for SBC at the −1st order are determined by the area bounded by the three sets of lines, including $R_{-1}\left(\frac{\lambda }{n_{i}p}=sin\theta_{i}+1\right)$, $T_{+1}\left(\frac{\lambda }{n_{i}p}=-sin\theta_{i}+\frac{n_{s}}{n_{i}}\right)\;$and $\;T_{-2}\left(\frac{\lambda }{n_{i}p}=\frac{1}{2}sin\theta_{i}+\frac{n_{s}}{2n_{i}}\right)$; with *n_s_* = 1.44964, and *n*_0_ = 1. Diffraction orders are only affected by the grating period, not the geometrical form of the grooves, according to Equation (2). Nevertheless, the amplitudes or diffraction efficiencies of the orders cannot be calculated by such a simple equation. The electromagnetic field inside the grating region, and hence the precise groove geometry, must be taken into account while calculating them. The angular separation between the diffraction orders is determined by the grating structure’s period, while the structure within a single grating period controls how the power is divided across the orders.

The propagation properties of the laser beams are characterized by a beam quality factor $M^{2}$. It shows the number of times the far-field divergence of a real beam is greater than the divergence of a perfect diffraction-limited Gaussian beam of the same size. The beam quality has improved when the beam quality factor $M^{2}$ is closer to 1 [12,26].

Assuming diffraction-limited output for individual lasers, incidence angles near Littrow, and wavefront-distortion-free diffraction from the grating, the combined beam quality varies with the single-channel $1/e^{2}$ linewidth Δλ as follows [12]:

(3) $$M^{2}=\sqrt{1+{\left(\frac{\pi \omega_{0}\Delta \lambda }{2p\lambda cos\theta_{i}}\right)}^{2}}$$

where: $\omega_{0}$ is the $1/e^{2}$ beam radius. For a SBC fiber array with total output power P, the peak irradiance on the grating is given by the following [12]:

(4) $$I_{peak}=\frac{2Pcos\theta_{i}}{\pi \omega_{1}^{2}}$$

where: $\omega_{1}$ is the $1/e^{2}$ beam radius for the diffracted beam. For a given value of Δλ and *p*, increasing $\theta_{i}$ will decrease $I_{peak}$ and the combined beam quality simultaneously.

The angular spread Δφ of a spectrum of order m between the wavelength λ and λ+Δλ can be obtained by differentiating the grating equation, assuming the incidence angle $\theta_{i}$ to be constant. The change D in diffraction angle per unit wavelength is, therefore, the following [26]:

(5) $$D=\frac{d\varphi }{d\lambda }=\frac{m}{pcos\theta_{m}}$$

The quantity D is called the angular dispersion. The substitution of Equation (1) into Equation (5) for the angular dispersion obtains the following [14]:

(6) $$D=\frac{d\varphi }{d\lambda }=\frac{sin\theta_{i}+sin\theta_{m}}{\lambda cos\theta_{m}}$$

When we consider the Littrow condition ($\theta_{i}=\theta_{m}$), Equation (6) will reduce to the following:

(7) $$D=\frac{d\varphi }{d\lambda }=\frac{2}{\lambda }tan\theta_{i}$$

Therefore, the incident angle at the Littrow condition can be determined by the following:

(8) $$\theta_{i}=\tan^{-1}\left(\frac{D\lambda }{2}\right)$$

The effective indices of the grating modes can be found by the following [28]:

(9) $$\cos\left(\alpha p\right)=F\left(n_{eff}^{2}\right)$$

where: *p* is the grating period and $\alpha =k_{0}sin\theta_{i}$, $k_{0}$ is a unit vector of incident wave.

For the TE polarization, the right-hand side of Equation (9) is given by the following [28,29]:

(10) $$F\left(n_{eff}^{2}\right)=\cos\left(\beta b\right)\cos\left(\gamma g\right)-\frac{\beta^{2}+\gamma^{2}}{2\beta \gamma }\sin\left(\beta b\right)\sin\left(\gamma g\right)$$

with: $\beta =k_{0}\sqrt{n_{b}^{2}-n_{eff}^{2}}$, $\gamma =k_{0}\sqrt{n_{g}^{2}-n_{eff}^{2}}$, b and g are the ridge and groove widths.

Because the grating is illuminated under Littrow mounting, the intersection of the illustrated functions$\;F\left(n_{eff}^{2}\right)$ and $\cos\left(\alpha p\right)=-1$ gives the effective indices of the modes that can be excited by the incident wave. Only two propagating modes have real effective indices; all higher-order modes are evanescent since their *n_eff_* is imaginary. The diffraction efficiency of the negative first order $\eta_{-1}$ can be expressed by the following:

(11) $$\eta_{-1}\left(h\right)=sin^{2}\left(\pi \frac{h}{\lambda }\left|n_{eff1}-n_{eff2}\right|\right)$$

Based on their calculation, the optimum height for the TE polarization can be determined by the following [28]:

(12) $$h_{max}^{TE}=\frac{\lambda }{2\left|n_{eff1}^{TE}-n_{eff1}^{TE}\right|}$$

Equation (12) implies that the incidence wavelength is directly proportional to the depth of the local surface relief structure. For the TM polarization, $F\left(n_{eff}^{2}\right)$ is in the following form [28]:

(13) $$F\left(n_{eff}^{2}\right)=\cos\left(\beta b\right)\cos\left(\gamma g\right)-\frac{\beta^{2}+\varepsilon_{b}^{2}\gamma^{2}}{2\varepsilon_{b}\beta \gamma }\sin\left(\beta b\right)\sin\left(\gamma g\right)$$

where: $\varepsilon_{b}=n_{b}^{2}$ is the dielectric permittivity of the substrate material.

To achieve a high diffraction efficiency for TM polarized light, it is also necessary to fulfill the phase condition Equation (12). The grating depth can be decided by solving Equations (11) and (13) for TE and TM polarizations. Therefore, the design must compromise between the contradictory tendencies. Moreover, no theoretical equation can estimate the number in an unpolarized case. For non-polarized light, according to Ref. [30], the average diffraction efficiency is defined as follows:

(14) $$\eta_{ave}=\frac{1}{2}\left(\eta_{TE}+\eta_{TM}\right)$$

In this study, an MDG structure is proposed for the high diffraction efficiency grating based on the finite element method by parameter scanning using COMSOL Multiphysics software version 5.5. Generally, diffraction gratings can be entirely characterized by the following set of parameters: duty cycle (f), grating period (p), and grating thickness (h). The width of the grating’s ridge b is represented by the product of f.p. The duty cycle value is optimized during the computation so that the grating has the most thorough structure. Besides, we need to be concerned about the light parameters, including polarization type (TE, TM, or unpolarized) incident angle $\theta_{i}$. Figure 2 shows a schematic of a basic rectangular diffraction grating. Between the gratings and the multilayer mirror, we add a matching layer. This layer not only serves as a transition layer between gratings and multilayer thin films but also improves the adhesion to the thin films, affecting the duration and operation efficiency of the whole system.

The combined efficiency of the SBC system is mostly determined by the grating diffraction efficiency [14]. The design of MDG for the SBC system of real beams is a procedure of optimizing grating parameters and desired parameters of individual lasers. In other words, the purpose of this approach is to minimize diffraction losses in the SBC system.

## 3. Results and Discussion

**Figure 3:**
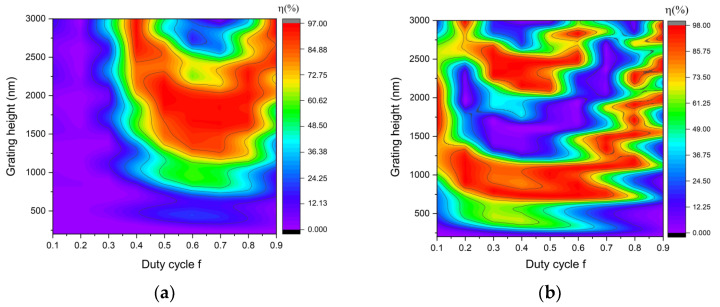

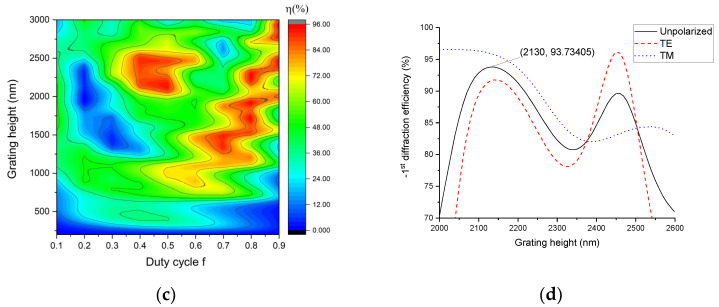
The −1st diffraction efficiency of rectangular−shaped grating for (**a**) TM polarization, (**b**) TE polarization, (**c**) unpolarized light, and (**d**) unpolarized light at f = 0.5.

Narrowing the parameter value is required to assess and determine the optimal parameters for grating. The incident angle near the Littrow line is selected based on the properties of Littrow mounting for a high diffraction efficiency. As a result, the Littrow criterion is met for this grating at an incidence angle of 44.43° (in the air). For the mentioned incidence angle, Equation (2) is used at 1064 nm, and the grating period is 760 nm. To demonstrate the increase in the diffraction efficiency, gold metal is employed as the reflective layer for the grating in the first stage. The performance of the grating depends on the polarization of the incident wave. Therefore, both a transverse electric (TE) and a transverse magnetic (TM) case are taken into account. The TE wave has the electric field component in the z-direction, out of the modeling xy-plane. For the TM wave, the electric field vector is pointing in the xy-plane and perpendicular to the direction of propagation, whereas the magnetic field has only a component in the z-direction. The simulations are run using a duty cycle range of f = 0.1~0.9. The obtained results from using the above parameters for the diffraction efficiency for TE, TM polarization are shown in Figure 3a,b. According to Equation (14), the diffraction efficiency of the grating for unpolarized light is shown in Figure 3c. Two duty cycle regions exhibit a substantial diffraction efficiency, one around 0.5 and the other around 0.7. The duty cycle f = 0.5 is chosen for its simplicity in design, ease of manufacturing, low cost, and availability in commercial production. As a result, Figure 3d shows the −1st diffraction efficiency of the rectangular-shaped grating for unpolarized light at f = 0.5. As shown in Figure 3d, if the grating may be utilized for non-polarized light, the grating depth is 2130 nm. The grating parameters are shown in Table 1.

**Figure 4:**
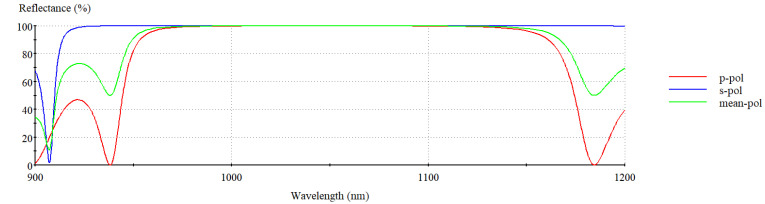
The reflectance of a dielectric multilayer was simulated using the Essential Macleod.

**Figure 5:**
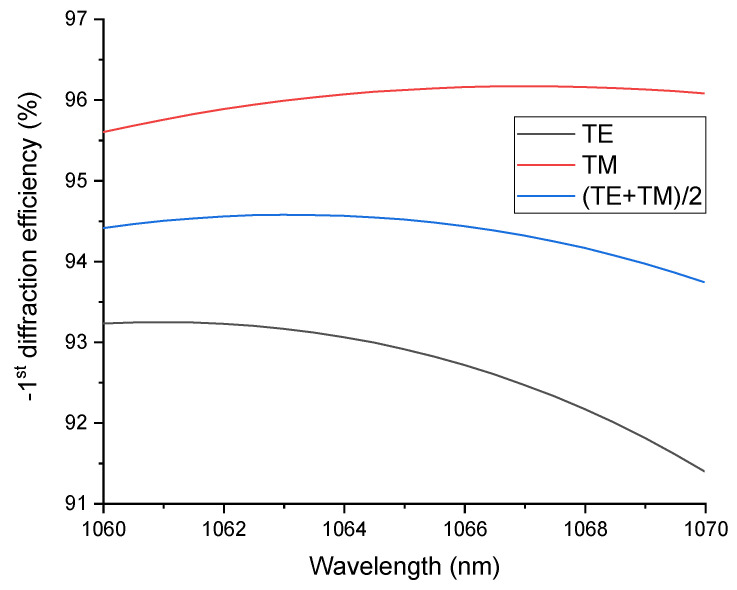
MDG diffraction efficiency at different wavelengths.

The downside of utilizing metal in the reflective layer, as previously stated, is absorption, which leads to a loss in the diffraction efficiency of the grating. The challenge is addressed by replacing the gold layer with a reflective dielectric multilayer according to the formula (HL)^13^ H, where H stands for the high refractive index of Ta_2_O_5_, and L stands for the low refractive index of SiO_2_. The LDT of coatings based on SiO_2_ can be up to 4.28 ± 0.07 J/cm^2^, which is the highest among other materials normally used for coatings [22]. This feature enables SiO_2_ in MDG to be able to withstand high-power laser radiation, making them ideal elements for high-power SBC. Ta_2_O_5_ is an important material for optical coatings as a result of its wide transparent spectrum, high refractive index, strong adhesion with substrates, and especially the Ta_2_O_5_/SiO_2_ multilayer high-reflection mirror, which has been widely investigated [31]. The reflectance of a dielectric multilayer is simulated using the Essential Macleod software version 11.4.585, as shown in Figure 4. The diffraction efficiency of the MDG is shown in Figure 5 at various wavelengths. We find that the unpolarized light’s diffraction efficiency was 94.5% at 1064 nm.

**Figure 6:**
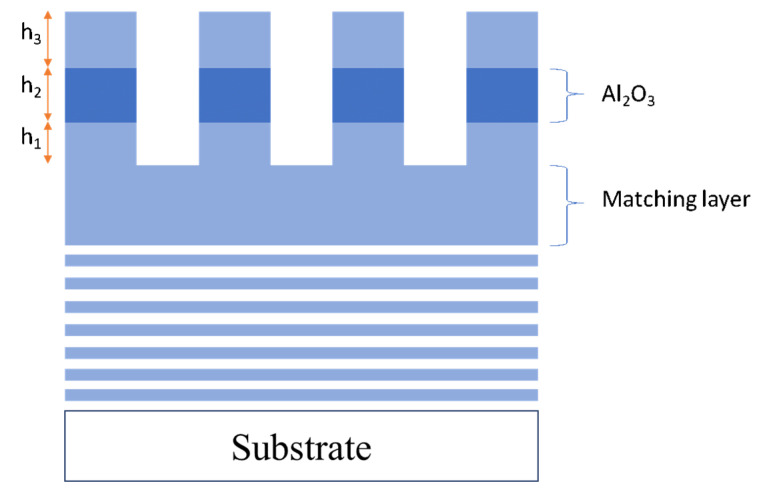
Schematic of three−layer MDG.

To date, three-layer gratings have greater diffraction efficiency than single-layer gratings. We discover that the diffraction performance may still be enhanced. Thus, in our work, an interlayer material is applied to the rectangular grating, as shown in Figure 6. The material must be different from SiO_2_ and have a high laser-induced damage threshold. With a reasonably high LDT of 2.52 J/cm^2^ [22], a high refractive index Al_2_O_3_ material is a good candidate for interlayer grating design. We merely alter the position and thickness of h_2_ in this case. The overall thickness of the grating is still 2130 nm, as shown in the above results.

**Figure 7:**
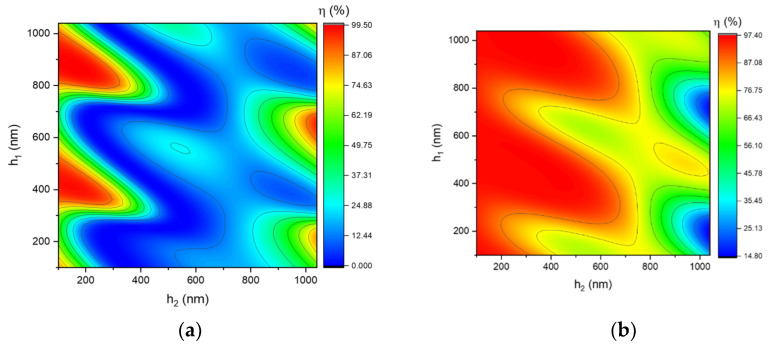

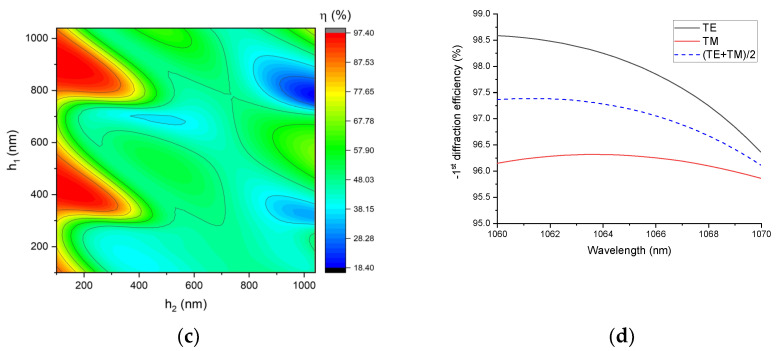
−1st diffraction efficiency of rectangular−shaped grating for (**a**) TE polarization and (**b**) TM polarization, (**c**) unpolarized light, and (**d**) diffraction efficiency of the optimum design.

**Figure 8:**
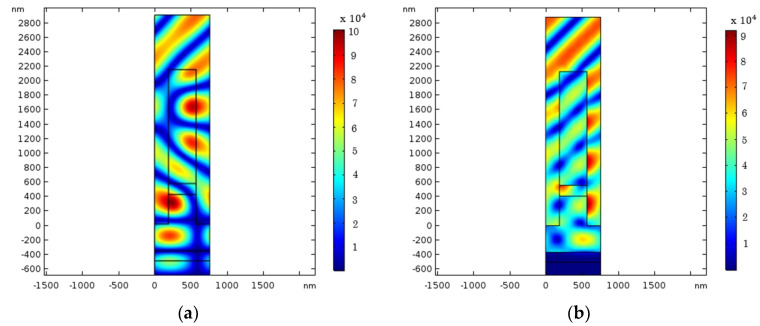
Normalized electric field distribution for (**a**) TE polarization and (**b**) TM polarization.

The location and height of the Al_2_O_3_ interlayer are simulated to determine the optimal position for diffraction efficiency and LDT. The TE-polarization and TM-polarization diffraction efficiency with different h_1_ and h_2_ are given in Figure 7a,b. Similarly, their means provide the −1st diffraction efficiency for unpolarized light in Figure 7c. According to this figure, two high diffraction efficiency regions are equivalent to two distinct h_2_ positions. The maximum electric field intensity (EFI) location is also critical because it will cause damage to the MDG. Hence, it is essential to determine the position of the added layer to prevent the maximum electric field focusing on this layer. The simulation range for LDT is confined to the two locations with the high diffraction efficiency described previously. This simulation shows the optimal height value of the grating structure for LDT is (h_1_, h_2_, h_3_) = (409 nm, 150 nm, and 1571 nm). As shown in Figure 8, the majority of the maximum EFI was spread on SiO_2_ layers in the TE polarization, whereas in the TM polarization, it was scattered outside of the grating. As a consequence, this grating design will function stably in a high-intensity laser system. The diffraction efficiency of the unpolarized light is 97.28% at 1064 nm, as shown in Figure 7d. This value is an increase of 2% compared with that of normal MDG. Table 2 shows the parameters of the three-layer MDG.

## 4. Conclusions

The grating of a rectangular-groove three-layer on a dielectric multilayer mirror is reported in this paper. A multilayer mirror ((HL)^13^ H; H: Ta_2_O_5_, L: SiO_2_), which replaces metal reflectors, provides a significant contribution to boosting the diffraction efficiency. Furthermore, using the optimal position for the Al_2_O_3_ interlayer of the grating plays a critical function in improving the diffraction efficiency and laser damage thresholds. The design of MDG is optimized for a 10 nm bandwidth non-polarized laser at 1064 nm with an absolute −first diffraction efficiency of 96.1–97.4%. Using appropriate materials and a well-designed structure, this grating also demonstrates a significant improvement in LDT through the electric field intensity distribution. The obtained results from the designed grating have a useful contribution to the development of new devices through the spectral beam combining method.

## Figures and Tables

**Table 1:** Rectangular shaped grating parameters.

Parameters	Value
Incident wavelength	1064 nm
Period	760 nm
Incident angle	44.43°
Duty cycle	0.5
Grating’s height	2130 nm
Reflective layer	Gold
The thickness of the matching layer (SiO_2_)	$366.99\;\mathrm{nm}\;\left(\delta /2\right)$
Refractive index of the substrate (Glass)	1.50664
Refractive index of SiO_2_	1.44964

**Table 2:** Parameter of the three-layer MDG.

Parameters	Value
Grating’s shape	Rectangular
Incident wavelength	1064 nm
Period	760 nm
Incident angle	44.43°
Duty cycle	0.5
Grating’s depth/height	2130 nm (409, 150, 1571)
The thickness of the matching layer (SiO_2_)	$366.99\;\mathrm{nm}\;\left(\delta /2\right)$
Refractive index of the substrate (Glass)	1.50664
Refractive index of SiO_2_	1.44964
Refractive index of Al_2_O_3_	1.6509
Refractive index of Ta_2_O_5_	2.10000
Reference wavelength	1164 nm
Reflective multilayer layer structure	Substrate| (HL)^13 H|Air H: Ta_2_O_5_ L: SiO_2_

## Data Availability

The data presented in this study are available in this article.
